# T-Helper1/T-Helper2 Cytokine Imbalance in the Iris of Patients with Glaucoma

**DOI:** 10.1371/journal.pone.0122184

**Published:** 2015-03-26

**Authors:** ManSin Wong, Ping Huang, Weiyi Li, Ying Li, Samuel S. Zhang, Chun Zhang

**Affiliations:** 1 Department of Ophthalmology, Peking University Third Hospital, Peking University Eye Center, Beijing, China; 2 Department of Ophthalmology, Renji Hospital, School of Medicine, Shanghai Jiao Tong University, Shanghai, China; 3 Department of Neural & Behavioral Sciences, Penn State University, Hershey, Pennsylvania, United States of America; Université Libre de Bruxelles, BELGIUM

## Abstract

The mechanistic study of glaucoma pathogenesis has shifted to seeking to understand the effects of immune responses on retinal ganglion cell damage and protection. Cytokines mediate the biological effects of the immune system, and our previous study revealed an imbalance of T-helper (Th) 1-derived and Th2-derived cytokines in the serum of patients with glaucoma. In this study, we collected irises from normal individuals and patients with primary open-angle closure (POAG) or chronic angle-closure glaucoma (CACG). We used real-time polymerase chain reaction (PCR) to measure the expression of Th1 (interleukin (IL)-2, interferon-gamma (IFN-γ)), Th2 (IL-4, IL-6, IL-10), and Th3 (transforming growth factor-beta (TGF-β)) cytokines. We then performed immunohistochemical staining to characterize the localization of the upregulated cytokines in iris cryosections. We observed an upward trend in the expression of IL-2 and IFN-γ and a downward trend in IL-6 expression in the iris of POAG and CACG patients. Expression of TGF-β also increased. Immunohistochemistry revealed that IL-2 expression in POAG and CACG patients was localized in the anterior surface of the blood vessel wall in the stroma of the iris, in the cytoplasm of some cells, in the anterior epithelium, and in the posterior pigment epithelium. These findings indicate that immune status differed between the iris tissues of POAG and CACG patients and those of normal individuals. A T-helper cytokine imbalance may modulate the immune microenvironment in glaucomatous eyes and thus influence optic neuropathy.

## Introduction

Glaucoma is one of the leading causes of blindness worldwide. It includes chronic neurodegenerative diseases of the optic nerve such as apoptosis of retinal ganglion cells (RGCs), progressive loss of optic nerve axons, and visual fields defects [1–2]. Increased intraocular pressure is considered a major risk factor [3–7], although numerous studies have suggested that glaucomatous neuropathy actually involves multiple factors, including impaired intraocular blood circulation [8–11], excitotoxic reactions caused by excess accumulation of glutamate [11–13], free radical production and oxidative stress [14–17], increased NO levels [18–20], and immunological factors.

The role of immunological factors in glaucoma has always been a major research topic. Becker et al [21]. first discovered plasma cells and immunoglobulins in trabecular biopsies of glaucomatous eyes, suggesting changes in humoral immunity. Later, a series of studies reported abnormalities in serum cytokines, antibodies, and the complement system. For example, higher levels of antibodies against small heat shock proteins were found in normal tension glaucoma patients, and several autoantibodies such as HSP70, anti-phosphatidylserine, γ-enolase, glycosaminoglycans, vimentin and retinal S-antigen were identified in glaucoma [22–26]. What’s more, the advances and understanding made in both animal models of glaucoma as well as in human glaucoma autopsy findings in the 90’s and 00’s suggests there is a fundamental role of the immune system in mediating neuronal cell death in glaucoma regardless of intraocular pressure [102–104].

Cytokines mediate immune and inflammatory responses in many situations and are widely involved in the process of glaucomatous optic neuropathy [27–36]. T-helper (Th) cells are the main source of cytokines and can be classified into subsets by their cytokine production profiles [37]. Th1 cells play a critical role in the regulation of cellular immunity by secreting interferon-gamma (IFN-γ), interleukin (IL)-2, IL-12, and tumor necrosis factor (TNF)-α. Th2 cells regulate humoral immunity by producing IL-4, IL-5, IL-6, IL-10, and IL-13. The concept of imbalanced Th subsets has been associated with a number of infectious and autoimmune diseases, allergies, immunodeficiencies, tumor progression, failed pregnancy, and graft rejection [38–41]. The concept of immune balance has recently been introduced to the study of central nervous system disorders [42–45]. A variety of cell types in the central nervous system produce and secrete cytokines, including neurons, microglia cells, stellate cells, and endothelial cells. Astrocytes and microglial cells play roles similar to those of Th1 and Th2 cells, respectively [46–48]. Glaucomatous optic neuropathy is a degenerative disease of the nervous system. Our previous clinical study showed significantly lower serum TNF-α in primary open-angle glaucoma (POAG) patients than in controls, while the levels of IL-4, IL-6, and IL-12p70 were significantly higher. The change in cytokine concentration is associated with the degree of optic neuropathy, suggesting that an imbalance of Th1/Th2 cytokines plays an important role in the mechanism of glaucomatous optic neuropathy [49–52].

To further explore the changes of cytokine profiles in the tissues of the eyeball under pathological conditions, we collected the iris during trabeculectomy and compared the levels of Th1 (IL-2, IFN-γ), Th2 (IL-4, IL-6, IL-10), Th3 (transforming growth factor-beta (TGF-β)) cytokines in POAG patients, CACG patients, and normal control subjects. A better understanding of the diverse roles of the immune system in glaucomatous optic nerve degeneration will facilitate the development of neuroprotective strategies in glaucoma.

## Methods

### Study subjects

This study was prospectively approved by the Clinical Ethics Committee of Peking University Third Hospital, and followed the principles suggested by the Declaration of Helsinki. In this cross-sectional study, consecutive patients (17 POAG and 18 CACG) who were admitted to the eye center of Peking University Third Hospital between March 2011 and April 2012 and were to undergo trabeculectomy were recruited. Prior to enrollment, all patients understood the methods and purposes of this study, and signed informed consent. Normal controls were 18 donated eyes selected from the eye bank at Peking University Third Hospital.

Subjects who satisfied the following criteria were enrolled: (1) a decrease in neuroretinal rim width to less than or equal to 0.1 of the cup-to-disc ratio or glaucomatous optic neuropathy diagnosed by an experienced glaucoma specialist; (2) a reliable and reproducible Humphrey visual field defect of greater than 5 dB in at least two points or greater than10 dB in a single point below age-specific normal threshold; (3) a mandatory diagnosis of exclusive NTG, POAG, or CACG. Glaucomatous optic neuropathy was determined as either cup/disc asymmetry between fellow eyes greater than 0.2, rim thinning, notching, excavation, or RNFL defect. CACG was defined as glaucoma (field defect, glaucomatous optic neuropathy as described above) with an occludable angle and elevated intraocular pressure (IOP) of more than 21 mmHg (Goldmann Applanation tonometer, Haag-Streit, Bern, Switzerland) on at least two separate occasions without history of acute attack. An occludable angle was classified when the posterior trabecular meshwork (usually pigmented) could not be seen over 270° or more of that angle without indentation. Glaucoma in the presence of an open angle was diagnosed as either POAG (with at least one recorded IOP more than 21 mmHg) or NTG (with an untreated IOP less than or equal to 21 mmHg at all diurnal phases). Normal eyes included eighteen donated eyes from the Eye Bank at Peking University Third Hospital.

Criteria for exclusion were as follows: (1)The treated eye had a history of trauma, surgery, or laser surgery. (2)In addition to minor cataracts, the treated eye was affected by other eye diseases such as diabetic retinopathy or age-related macular degeneration. (3)The treated eye was affected by immune-related eye diseases such as uveitis. (4)The patient suffered from systemic immune disease or autoimmune disease. (5)The patient had a long history of smoking and medication; excess alcohol consumption; serious heart, liver, and kidney diseases; or blood disease. (6)Signs and symptoms of infection were observed within 6 months before surgery.

### Sample collection

All trabeculectomy procedures were performed by Dr. Zhang or Dr. Huang at the Eye Center of Peking University Third Hospital. Surgeries were performed according to classic trabeculectomy procedures and resected iris tissues were collected during peripheral iridectomy. Normal control eyes were freshly donated whole globes from the Eye Bank at Peking University Third Hospital. Iris tissues of comparable size were resected from the peripheral iris after removing the transparent cornea.

Freshly cut iris tissues were divided into two parts. One part was immediately preserved in RNAlater RNA Stabilization Reagent (Qiagen, Hilden, Germany) for cytokine level determination. The volume of RNAlater was at least 10 times the volume of the tissue specimen. The samples were first stored at 4°C for 24 h, followed by storage at −80°C. The remaining samples were immediately embedded in opti-mum cutting temperature compound (Sakura, USA), frozen in liquid nitrogen for 10 s, and stored at −80°C for cryosection and immunohistochemical staining.

### Quantitative real-time polymerase chain reaction (PCR)

Total mRNA from each sample was extracted using TRIzol reagent (Invitrogen, Carlsbad, USA), according to the manufacturer’s protocol. cDNA was synthesized with SuperScript III Transcript (Invitrogen). This was followed by PCR amplification with Power SYBR Green PCR Master Mix (Invitrogen). Cycling conditions were as follows; denaturation at 95°C for 2 min followed by 40 cycles at 95°C for 10 s, 58°C for 30 s, and melting at 60°C in a 7500Fast (Applied Biosystems, Foster city, CA, USA). The cycle threshold was calculated using the system software, and the results were analyzed using the comparative cycle threshold method. Table 1 shows the primers used for PCR amplification.

**Table 1:** Primers used for PCR amplification of cytokines.

Cytokines	Forward Primer	Reverse Primer
IL2	5′-AACCTCAACTCCTGCCACAA-3′	5′-GCATCCTGGTGAGTTTGGGA-3′
IL4	5′-AGCAGTTCCACAGGCACAAG-3′	5′-ACTCTGGTTGGCTTCCTTCAC-3′
IL6	5′-TTCGGTCCAGTTGCCTTCTC-3′	5′-TGAGATGCCGTCGAGGATG-3′
IL10	5′-GAGGAAAAAAAATGTTCTTTGGGGA-3′	5′-GGGGCTCCCTGGTTTCTCTTCCTAA-3′
IFN-γ	5′-GGCCAGGAATCAGAATCAG-3′	5′-CAGTGTCACTATGGTGCTTGTC-3′
TGF-β	5′-ATTCCTGGCGATACCTCAGC-3′	5′-CTCAACCACTGCCGCACAA-3′
GAPDH	5′-AGAAGGCTGGGGCTCATTTG-3′	5′-AGGGGCCATCCACAGTCTTC-3′

### Immunohistochemistry

Cryostat sections of the iris (7-μm thick) were mounted onto poly-l-lysine-coated slides. Slides were washed three times with 0.01% PBS buffer for 5 min, followed by 0.5% potassium permanganate for 5 min in the dark for depigmentation, and then with tap water. Slides were immersed in 2% oxalic acid for 5 s and washed with tap water. The slides were blocked in blocking serum (5% bovine serum albumin in PBS) for 1 h at room temperature and then incubated with rabbit anti-IL-2 (1:250, Abcam, Cambridge, UK), rabbit anti-IFN-γ(1:100, Cell Signaling Technology, Danvers, USA), or mouse anti-TGF-β1 (1:250, Abcam) overnight at 4°C. After three 5-min washes in PBS, the slides were incubated with the appropriate secondary antibodies, namely Peroxidase-conjugated AffiniPure goat anti-rabbit IgG (H+L) (1:1000, ASGB-BIO, Beijing, China) and Peroxidase-conjugated AffiniPure goat anti-mouse IgG (H+L) (1:1000, ASGB-BIO) at 37°C for 45 min. For signal detection, the sections were incubated with the ready-to-use 3-aminoethylcarbazole (AEC) substrate-chromogen solution (ASGB-BIO) for 5 min and then washed with distilled water. Finally, sections were counterstained with hematoxylin for 1 min, followed by washing with distilled water and coating with a thin layer of immersion oil. Second antibody alone was used as blank controls.

Images were visualized and captured by LeicaDM4000B microscopy (Wetziar, German). All the images were captured with the same exposure brightness and exposure time under 20 magnification.

### Statistical analysis

Statistical analysis was performed in SPSS17.0 software. Differences as per gender were analyzed by Pearson’s chi-squared test; the number of preoperational eye drops used was analyzed by the Mann–Whitney U test; age, preoperational intraocular pressure, and average visual field deterioration were analyzed by Student’s *t*-test. Differences in cytokine levels between groups were analyzed by ANOVA Least Significant Difference test. A *P* value ≤ 0.05 was considered statistically significant, and a *P* value ≤ 0.01 was considered highly statistically significant.

## Results

### Sample evaluation

Seventeen patients (10 male and 7 female) were included in the POAG group and 18 patients (7 male and 11 female) were included in the CACG group. No significant difference was found in the gender composition of these groups. Average age was 56.06 ± 13.83 years (POAG) and 63.83 ± 8.70 years (CACG); the average number of eye drops used before surgery was 2.5 ± 0.80 and 2.50 ± 0.85; the preoperational intraocular pressure was 25.05 ± 4.07 mmHg and 27.50 ± 6.14 mmHg; none of these parameters differed significantly between groups. The mean defect (MD) of visual field in the POAG and CACG groups was 0.90 ± 4.60 and 15.13 ± 7.53, respectively (P = 0.010) (Table 2).

**Table 2:** Patient characteristics.

Diagnosis	POAG (n = 17)	CACG (n = 18)	*P* value (2-sided)
Gender (male/female)	10/7	7/11	0.238
Age (years)	56.06 ± 13.83	63.83 ± 8.70	0.058
Medications	2.50 ± 0.80	2.50 ± 0.85	0.910
Intraocular pressure (mmHg)	25.05 ± 4.07	27.50 ± 6.14	0.178
Mean defect	20.90 ± 4.60	15.13 ± 7.53	0.010

### Increasing Th1 and Th3 cytokines in glaucomatous iris by quantitative real-time PCR

The melting curves for the cytokine and GAPDH reactions showed single peaks, suggesting the reactions were specific and the results of the fluorescence quantitation were accurate and reliable. The original real-time PCR data were calculated using the F = 2^–ΔΔCt^ formula to quantify relative expression levels.

The mean relative expression level of IL-2 was 0.361±0.068 in POAG group, 0.350±0.063 in CACG group and 0.181±0.032 in normal controls respectively. Statistical analysis showed that the expression of IL-2 increased in the POAG (1.99-fold) and CACG (1.93-fold) groups versus the controls; the difference between the POAG and CACG groups was not significant (POAG vs. controls, *P* = 0.025; CACG vs. controls, *P* = 0.035; POAG vs. CACG, *P* = 0.893).

The mean relative expression level of IFN-γwas 0.359±0.071 in POAG group, 0.463±0.081 in CACG group and 0.206±0.029 in normal controls respectively. Expression of IFN-γ increased 2.24-fold in the CACG group versus the controls; expression in the POAG group trended upward and was 1.74-fold greater than in the controls. The difference between the POAG and CACG groups was not significant (POAG vs. controls, *P* = 0.101; CACG vs. controls, *P* = 0.007; POAG vs. CACG, *P* = 0.264).

**Fig 1:**
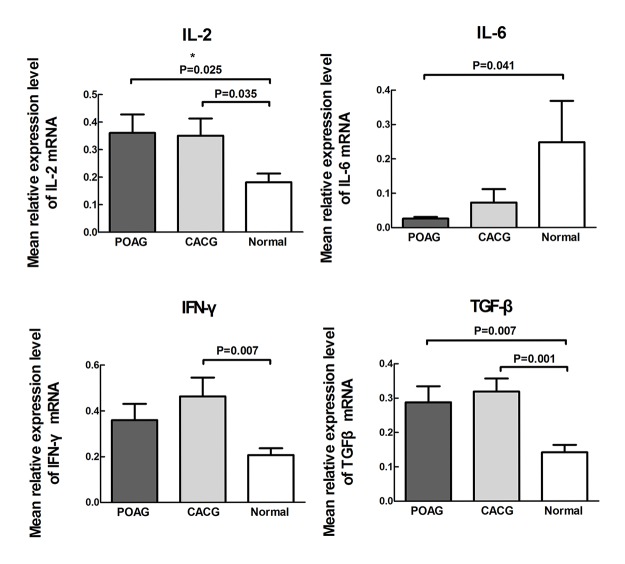
Interleukin (IL)-2, IL-6, interferon-gamma (IFN-γ), and transforming growth factor-beta (TGF-β) in the irises of primary open-angle glaucoma (POAG) and chronic angle-closure glaucoma (CACG) patients and control subjects. Differences between groups were analyzed by ANOVA Least Significant Difference test. *P* value ≤ 0.05 was considered statistically significant. Mean concentrations of IL-2, IFN-γ, and TGF-β showed an upward trend in the iris of POAG and CACG patients. A downward trend of IL-6 was observed in the irises of POAG and CACG patients.

The mean relative expression level of TGF-βwas 0.288±0.046 in POAG group, 0.319±0.038 in CACG group and 0.142±0.021 in normal controls respectively. Compared to the normal group, TGF-β expression increased in the POAG (2.03-fold) and CACG (2.25-fold) groups versus the controls; the difference between the POAG and CACG groups was not statistically significant (POAG vs. controls, *P* = 0.007; CACG vs. controls, *P* = 0.001; POAG vs. CACG, *P* = 0.549). (Fig. 1)

### Decreasing Th2 cytokines in glaucomatous iris by quantitative real-time PCR

As the same method used for examination of Th1 and 3 cytokines in iris, we also observed the levels of Th2 cytokines, IL-4, IL-6, and IL-10.

The mean relative expression level of IL-6 was 0.026±0.005 in POAG group, 0.073±0.039 in CACG group and 0.248±0.120 in normal controls respectively. Compared to the normal control, IL-6 expression decreased in the POAG group. Expression in the controls was 9.47-fold greater than in the POAG group. Expression trended downward in the CACG group; expression in the controls was 3.41-fold greater than in the CACG group. The difference between the POAG and CACG groups was not significant (POAG vs. controls, *P* = 0.041; CACG vs. controls, *P* = 0.100; POAG vs. CACG, *P* = 0.662).

IL-4 and IL-10 were not detectable (most samples yielded no amplification), suggesting that protein expression of these cytokines was very low in the iris.

### Variety in protein expression of cytokines in iris by Immunohistochemistry

Consistent with the real-time PCR results, expression levels of IL-2, IFN-γ, and TGF-β in the iris of glaucoma patients increased or showed an upward trend. Further immunohistochemical staining was performed to determine IL-2, IFN-γ, and TGF-β localization.

**Fig 2:**
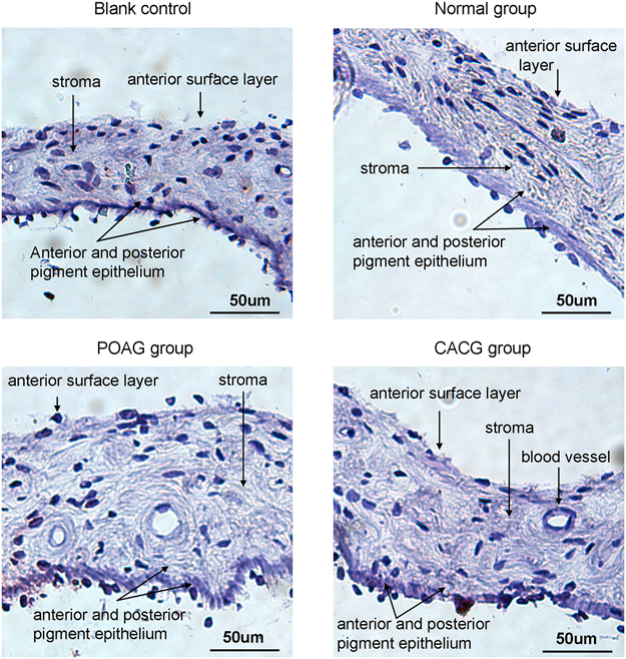
Interferon-gamma (IFN-γ) status in sections of human iris, as shown by AEC staining. Results for IFN-γ were negative in all examined iris sections. Scale bar, 50 μm

**Fig 3:**
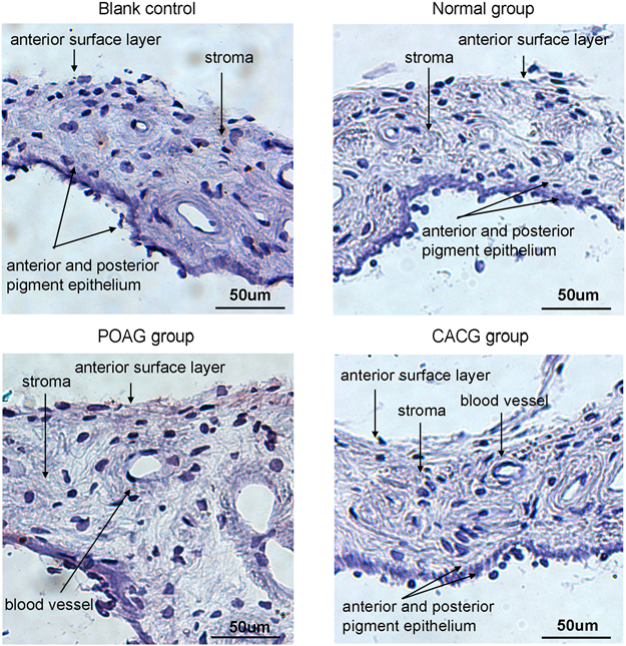
Transforming growth factor-beta (TGF-β) status in sections of human iris, as shown by AEC staining. TGF-β results were negative in all examined iris sections. Scale bar, 50 μm

IFN-γ was negative in all examined sections, suggesting that the trace expression of these two cytokines was below the detection limit (Fig. 2). A light positive staining of TGF-β was observed in the anterior surface layer of iris sample from POAG patient, but not in the samples from CACG patients and normal controls (Fig. 3).

**Fig 4:**
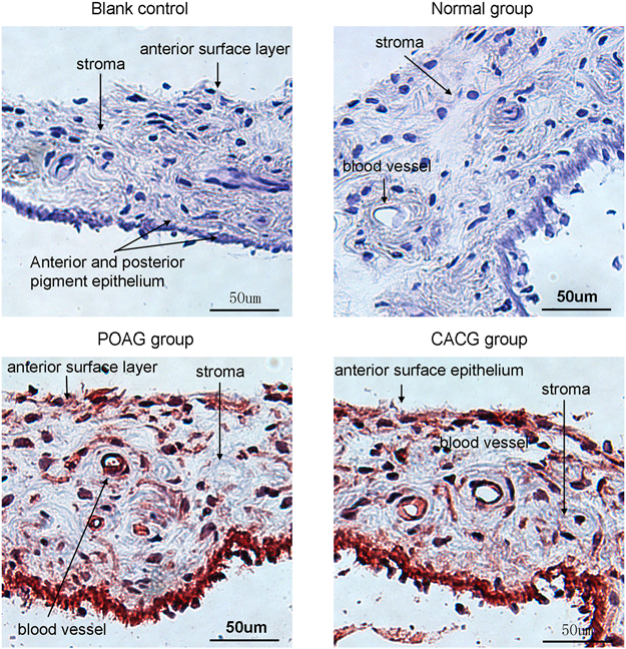
Interleukin (IL)-2 status in sections of human iris, as shown by AEC staining. IL-2 results were negative in the control subjects and positive in the primary open-angle glaucoma (POAG) and chronic angle-closure glaucoma (CACG) patients. Positive staining was localized to the anterior surface of the iris, the blood vessel wall in the stroma, the cytoplasm of some cells, and the posterior pigment epithelium. Scale bar, 50 μm

IL-2 was negative in the controls and positive in the POAG and CACG groups. Positive staining was localized to the anterior surface of the iris, the blood vessel wall in the stroma, in the cytoplasm of some cells, and in the posterior pigment epithelium (Fig. 4).

## Discussion

This was the first study to quantify and localize the expression of cytokines in the iris of glaucoma patients. Iris consists of two layers, the anterior pigmented fibrovascular stroma and posterior pigmented epithelial cells, as one of the major structures of anterior chamber. The changes of cytokine profiles under glaucomatous condition in iris could represent at least part of immunological alteration in glaucoma pathogenesis. We used real-time PCR to measure relative expression of IL-2, IL-4, IL-6, IL-10, IFN-γ, and TGF-β in the iris of POAG and CACG patients and normal individuals. Immunohistochemical staining was used to localize the elevated cytokines in iris cryosections. In the iris of glaucoma patients, expression of Th1 cytokines (IL-2 and IFN-γ) increased, Th2 cytokine IL-6 decreased, and TGF-β increased. Immunohistochemistry revealed IL-2 expression in the anterior surface of the iris, the blood vessel wall in the stroma, in the cytoplasm of some cells, and in the posterior pigment epithelium, suggesting that immune levels in the iris of glaucoma patients are different from those in the iris of normal individuals. Imbalances in Th cytokine subsets may regulate the immune microenvironment in a glaucomatous eye and play an important role in optic nerve damage in the retina.

Since human body specimens are valuable and difficult to obtain, only a few studies on cytokine expression levels in human glaucoma have been reported, and most were performed on the peripheral blood, aqueous humor, or the retinal tissues [27–36]. Currently the concept of immune balance has been introduced to the research on central nervous system disorders. The central nervous system itself has a mechanism to maintain immune balance and participate in the immune response [43–45]. A variety of cells in the central nervous system, including neurons, microglia cells, stellate cells, and endothelial cells, can produce and secrete different cytokines. Astrocytes and microglia cells, in the retina and the optic nerve, play roles similar to Th1 and Th2 cells, respectively [46–48]. Glaucomatous optic neuropathy is a degenerative disease of the nervous system. Our previous clinical study showed that serum TNF-α level in the POAG patients was significantly lower than that of control, and the levels of IL-4, IL-6, and IL-12p70 were significantly higher than that of control. Change of cytokine concentration is associated with the degree of optic neuropathy, suggesting that imbalance of Th1/Th2 cytokines plays an important role in the mechanism of glaucomatous optic neuropathy [49–52].

No study on cytokine levels in uveal tissues of glaucoma patients has been reported yet. However, the uvea is the tissue in the eye that is rich in blood vessels, lymphocytes, and monocytes. Aggregation of lymphocytes in the uvea has similar functions to those of lymph nodes, as it allows eliciting any type of allergic reaction and synthesizing IgG. Therefore, the uvea may reflect immune status in the local microenvironment of the eye. In this study, resected uveal tissues (irises) were collected after trabeculectomy to study changes in cytokine levels in the local microenvironment of the eyeball.

Th1 cells mainly secrete inflammatory cytokines such as IL-2, IL-12, IL-27, IFN-γ, and TNF. Th1 cytokines are associated with nerve damage [53–64]. In this study, IL-2 and IFN-γ expression increased in the iris of POAG and CACG patients, suggesting that their induction may be involved in the mechanism of glaucomatous optic neuropathy. IL-2, also called T-cell growth factor (TCGF), is expressed in a majority of T cells and promotes cytotoxic T lymphocyte and natural killer (NK) cell differentiation [37]. Yang et al.[29] discovered that the expression levels of soluble IL-2 receptor (sIL-2R) significantly increased in the peripheral blood of POAG and NTG patients. Studies in a mouse model of experimental autoimmune uveoretinitis showed that increased IL-2 is closely associated with RGC damage [53]. The main function of interferon (IFN)-γ is to activate NK and M∮ cells and to promote Th0 cell differentiation into Th1 cells [37]. Chua et al.[31] reported increased IFN-γ in the aqueous humor of POAG and PACG patients. Zhou et al.[32] found that in a rat model of chronic ocular hypertension, rats that received polypeptide Copolymer-1 immunization and retinal stem cell transplantation in the vitreous cavity showed reduced RGC apoptosis. IFN-γ levels were also downregulated in the aqueous humor and peripheral blood, suggesting that low levels of IFN-γ help prevent RGS apoptosis.

Th2 cells mainly secrete protective cytokines such as IL-4, IL-6, and IL-10. Th2 cytokines are associated with nerve protection [65–67]. In this study, IL-4 and IL-10 transcripts were not detected due to their low expression in iris tissues, and IL-6 level was significantly reduced in the iris of glaucoma patients, suggesting its downregulation may be involved in the mechanism of glaucomatous optic neuropathy. IL-6 is a neuroprotective factor in many neurodegenerative diseases [68–69]. Animal studies also show that in the rat retina, exogenous application of IL-6 after ischemia-reperfusion injury could reduce RGC apoptosis by 50%–70% [70]. The IL-6 receptor system consists of two molecules, IL-6Ra and gp130 [71]. IL-6 binding to gp130 activates JAK tyrosine kinase and then signal transducer and activator of transcription 3 (STAT3). Activation of STAT3 triggers expression of its target genes, BCL-2 and BCL-XL, which inhibit apoptosis. Studies have shown that STAT3 activation is necessary for RGC survival and provides a powerful neuroprotective effect [72]. Sanchez et al.[73] studied the neuroprotective mechanism of endogenous IL-6 and found that after ischemia-reperfusion injury, IL-6 and IL-6R were upregulated in the retina, whereas no significant change in gp130 was observed. Adding gp130 antibodies aggravated injury and increased the number of apoptotic RGCs. In our study, endogenous IL-6 decreased; therefore, STAT3 activation was prompted by a decrease in IL-6-gp130-JAK-STAT3 signal transduction, increasing the number of apoptotic RGCs.

The results of this study are not entirely consistent with the results of our previous clinical study on serum cytokines in POAG patients. Our previous study suggested IL-2 and IFN-γ levels do not differ between POAG patients and normal controls, and the levels of IL-4 and IL-6 were higher in POAG patients than in the control group [74]. Possible reasons for the difference are as follows: 1. Under normal conditions, antigens or immunogenic substances cannot pass through the blood-retinal barrier to enter the retina. The uvea is the only tissue in the eye that is rich in blood vessels, lymphocytes, and monocytes, has functions similar to those of lymph nodes, and is considered the immune activity center of the eyeball. In this study, cytokine measurements were made directly in the iris tissue, which is a better reflection of the true immune status in the eye microenvironment than peripheral blood is. 2. Cytokines have “trace amount” and “instant secretion” characteristics. “Trace amount” refers to extremely low cytokine concentrations in normal serum. Target cytokine-secreting cells are not distant, but are adjacent to the secreting cells or are the secreting cells themselves; therefore, cytokines do not require blood or body fluid for transport, and only need to ensure that effective local concentrations are achieved. Thus, direct examination of a patient’s iris may be better than examining the serum when exploring immune balance. “Instant secretion” refers to cytokine expression levels being usually low, with rapid, massive expression only occurring in response to immunostimulation. Secretion quickly stops when approaching completion of the response. The half-life of cytokines is extremely short, and expression levels change at different times, rendering clinical detection and data analysis difficult; this may also explain our inconsistent results [37].

This study also showed increased TGF-β mRNA expression levels in the iris of POAG and CACG patients, suggesting that TGF-β expression may be associated with glaucomatous optic neuropathy. Numerous studies have shown an increase of TGF in the aqueous humor of POAG patients [75–85]. High levels of TGF are directly involved in the pathogenesis of POAG [86–89]. Recent studies have focused on the effect of TGF on the trabecular meshwork and suggest that the abnormal expression of TGF causes decreased migration, proliferation, and phagocytosis of trabecular meshwork cells, resulting in increased resistance to aqueous humor outflow and intraocular pressure. TGF also induces transglutaminase expression in the trabecular meshwork, resulting in increased irreversible cross-linking of extracellular matrix proteins and increased resistance to aqueous humor outflow [90–94]. In addition to the impact on the trabecular meshwork, studies have also suggested TGF involvement in structural changes of the optic nerve head. Pena et al.[95] discovered that in the human glaucomatous optic nerve, TGF-β2 immunoreactivity is 70–100 times that of the normal optic nerve. Fukuchi et al.[96] induced chronic ocular hypertension injury in monkeys and found that TGF-β1 and TGF-β2 expression levels increased in glial cells surrounding the optic nerve head. Secretion of extracellular matrix (ECM) protein also increased, contributing to structural remodeling of the optic nerve head.

In this study, we used immunohistochemistry to observe the localization of highly expressed cytokines in iris cryosections. IFN-γ and TGF-β results were negative in all examined sections, perhaps because the trace expression of these cytokines was below the detection limit. IL-2 was expressed in the anterior surface layer, the blood vessel wall in the stroma, the cytoplasm of some cells, and the posterior pigment epithelium, suggesting weakened function of perivascular barriers in glaucoma patients, allowing cytokines to move from the blood into tissues, which is consistent with previous reports suggesting decreased function of perivascular barriers in glaucoma [97–99].

To our knowledge, this is also the first report of cytokine levels in CACG patients. It is generally believed that the pathogenic mechanisms of open-angle and angle-closure glaucoma are different, and that ocular pressure-independent factors may be more involved in the pathogenesis of optic neuropathy in POAG and normal tension glaucoma. Past studies have largely been based on the data from POAG and normal tension glaucoma. This study included both POAG and CACG patients and revealed showed consistent upward or downward trends in cytokine mRNA levels in the POAG and CACG groups. In addition, no significant difference in cytokine mRNA levels was observed between the POAG and CACG groups. Immunohistochemistry also showed consistent quality and localization of cytokine expression between these two groups. Consider the following: if previous studies suggested that immunological factors are involved in the pathogenesis of POAG and the mechanism of neuropathy, then the results of the current study may also suggest that immunological factors are involved in the pathogenesis of CACG, and cannot be ignored in the progression of optic neuropathy. However, it is also possible that POAG and CACG patients who received trabeculectomy were chronic glaucoma patients unresponsive to medication and laser treatments to prevent optic neuropathy progression. Thus, the changing cytokine levels may result from persistent optic neuropathy in glaucoma patients, and the observed late-stage expression.

Our results suggest that in the iris of glaucoma patients, Th1 cytokine expression levels increase, Th2 cytokine expression levels decrease, and the expression level of TGF-β increases, suggesting a Th1/Th2 cytokine imbalance in glaucomatous eyes. Our findings are similar to some traditionally acknowledged autoimmune diseases such as rheumatoid arthritis, which also showed an increase of Th1 cytokines expression [102]. There are some shortcomings of the current study. Due to limited iris specimens and trace expression of cytokines, cytokine levels were below the detection limits of many immunohistological methods. In this study, we chose to use highly sensitive real-time PCR molecular biology detection method. However, its disadvantage is that it detects cytokine mRNA expression levels, and cannot reflect translation, protein modification, or even biological function, nor is it a direct reflection of cytokine concentrations or activity.

There is no evidence indicating whether cytokine alterations in the iris are directly correlated to cytokine levels in the retina, but future experimental studies will provide clues to the underlying mechanisms of cytokine responses in retinal ganglion cells. The signaling pathways also remain unknown and will be the subject of future research. Cytokine knockout techniques have been widely used in immune intervention research, making it possible to study the effect of a single cytokine on an optic nerve [100–101]. The unique immune microenvironment and immune response characteristics in the eye may lead to the development of unique immunotherapies. In-depth studies of the immune mechanisms of glaucoma may reveal previously undiscovered immune mysteries in the eye, supporting the development of immunotherapeutic drugs and treatments for glaucomatous optic neuropathy and opening new paths to glaucoma treatment and optic nerve protection.
